# Challenges in Acanthamoeba Keratitis: A Review

**DOI:** 10.3390/jcm10050942

**Published:** 2021-03-01

**Authors:** Giuseppe Varacalli, Antonio Di Zazzo, Tommaso Mori, Thomas H. Dohlman, Sara Spelta, Marco Coassin, Stefano Bonini

**Affiliations:** 1 Ophthalmology Operative Complex Unit, Campus Bio-Medico University Hospital, 00128 Rome, Italy; g.varacalli@unicampus.it (G.V.); antoniodizazzo@gmail.com (A.D.Z.); t.mori@unicampus.it (T.M.); s.spelta@unicampus.it (S.S.); s.bonini@unicampus.it (S.B.); 2 Massachusetts Eye and Ear, Department of Ophthalmology, Harvard Medical School, 243 Charles Street, Boston, MA 02114, USA; thomas_dohlman@meei.harvard.edu

**Keywords:** Acanthamoeba keratitis, in vivo confocal microscopy, misdiagnosis, prognosis, therapy

## Abstract

To review challenges in the diagnosis and management of Acanthamoeba keratitis (AK), along with prognostic factors, in order to help ophthalmologists avoid misdiagnosis, protracted treatment periods, and long-term negative sequelae, with an overarching goal of improving patient outcomes and quality of life, we examined AK studies published between January 1998 and December 2019. All manuscripts describing clinical manifestations, diagnosis, treatment, prognosis, and challenges in short- and long-term management were included. The diagnosis of AK is often challenging. An increased time between symptom onset and the initiation of appropriate therapy is associated with poorer visual outcomes. The timely initiation of standardized antiamoebic therapies improves visual outcomes, decreases the duration of treatment, and reduces the chances of needing surgical intervention. In clinical practice, AK diagnosis is often missed or delayed, leading to poorer final visual outcomes and a negative impact on patient morbidity and quality of life.

## 1. Introduction

In humans, *Acanthamoeba* spp. are responsible for a painful and sight-threatening disease that has a significant negative impact on patient quality of life. The diagnosis of Acanthamoeba keratitis (AK) is often delayed and patients frequently experience protracted treatment courses that can require surgical intervention in an attempt to restore corneal integrity and health [1,2,3,4].

AK is relatively rare among corneal infections, with an estimated prevalence of 1–9 cases per 100,000. However, in Western countries, the incidence of AK has been steadily rising in direct correlation with contact lens wearing, which is the predominant risk factor [3,5,6,7,8]. Approximately 93% of all cases of AK are reported to occur in contact lens wearers [9,10,11,12]. Poor contact lens hygiene, such as overnight wear or wearing lenses during swimming and showering, is a known risk factor for AK [4,6,12,13,14]; there is also an increased risk amongst monthly disposable contact lens wearers [6]. In addition, orthokeratology is considered a major risk factor for AK, with an annual incidence of 7.7 cases per 10,000 [8].

*Acanthamoeba* spp. is a free-living, ubiquitous protozoan that is commonly found in freshwater and soil [7,12,15]. It exists in two forms: motile, which replicate trophozoites, and dormant cysts, which have minimal metabolic activity and are much more resistant to adverse conditions, such as extremes in temperature, dryness, and pH, as well as antiamoebic drugs [7,16,17,18]. 

Despite new diagnostic modalities and protocols, the diagnosis of AK is frequently missed and/or delayed, critically affecting patient prognosis, outcomes, and ultimately, quality of life. In fact, a delayed diagnosis often allows for deeper corneal involvement with severe sequelae requiring more intensive treatment, including surgery. The aim of the current review is to synthesize the clinical presentation and timing of an AK diagnosis with long-term outcomes and to review currently available diagnostic and therapeutic options for this condition.

## 2. Materials and Methods

A literature search of studies on AK that were published between January 1998 and August 2019 available on PubMed, Web of Science, and Google scholar was made without any language constraints but limited to human studies. All published peer-reviewed randomized clinical trials, case series, and case reports about clinical, diagnosis, treatment, and prognosis aspects of AK were evaluated. A total of 78 manuscripts were screened and 51 were included based on their subject matter, which included information regarding AK diagnosis, prognosis, treatment, and short- and long-term management challenges. 

## 3. Results

**Figure 1:**
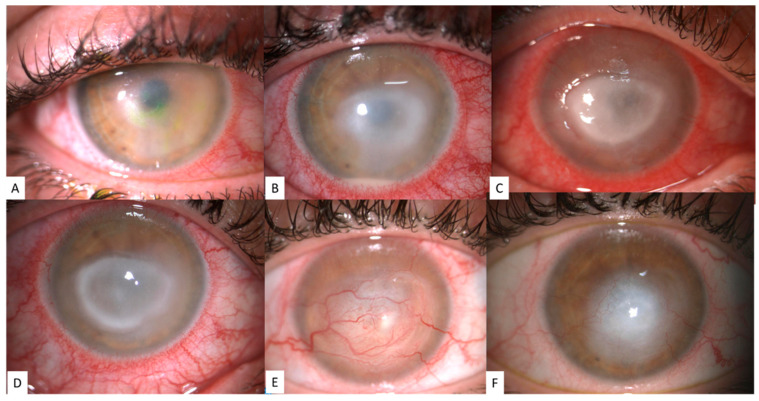
Representative images of the clinical evolution of Acanthamoeba keratitis (AK): early phase AK showing epithelial keratopathy (**A**, stage I) [3], stromal involvement and sterile hypopyon (**B**, stage II) [15], epithelial defect and ring stromal infiltrate (**C**,**D**, stage III) [11], and corneal scarring with deep and superficial neovascularization (**E**,**F**).

The diagnosis of AK originates from the patient’s history, patient’s presentation, and clinical suspicion. Symptoms frequently seen in AK include severe ocular pain, associated tearing, redness, photophobia, and decreased vision [1,3,4,6,7,11,12,19,20,21]. Generally, patients will have a unilateral presentation, but in up to 7.5% of cases, the presentation may be bilateral (Figure 1) [7,11]. Early clinical signs of infection include limbitis (95% of cases), perineural infiltrates (57% of cases), and punctate keratitis (46% of cases), as well as less common clinical signs, such as pseudodendrites and epithelial or subepithelial infiltrates; after two months, the collection of common clinical signs continues to include limbitis (96% of cases), as well as a ring infiltrate (or “Wessely immune ring,” 83% of cases), epithelial defects (75% of cases), and uveitis (79% of cases) [3,6,11,15,21,22]. 

**Figure 2:**
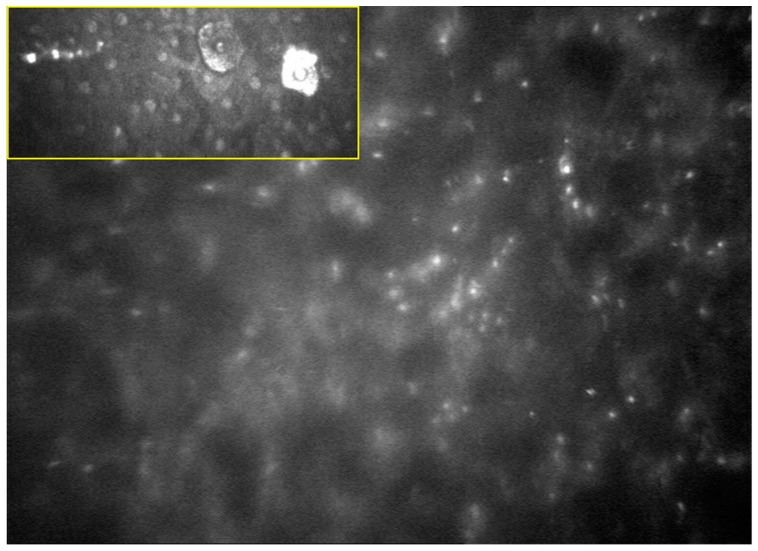
In vivo confocal microscopy image of Acanthamoeba keratitis involving superficial and deeper corneal layers. The typical “starry sky” appearance is depicted: *Acanthamoeba* spp. cysts appear as oval or round, double-walled, highly refractile structures with a polygonal inner wall and a total size of 12–25 microns [23].

Ophthalmologists now have access to several adjunct procedures and imaging modalities to assist in the diagnosis of AK. Cytological staining using giemsa or calcofluor white after corneal scraping, in vivo confocal microscopy (IVCM), a culture of corneal scrapings, polymerase chain reaction (PCR), and histology of corneal biopsies can all serve to help confirm clinical suspicions [4,11,23]. In particular, confocal microscopy, with a sensitivity and specificity of >90% [11,17,24,25], is an invaluable technique in the early investigation of patients with clinical presentations that potentially indicate AK [6,8]. IVCM provides rapid, detailed images of the corneal epithelium, stroma, and endothelium, and permits in vivo identification and observation of microorganisms without using stains, dyes, or tissue fixation [26]. *Acanthamoeba* spp. is difficult to grow or detect in vitro; therefore IVCM is a helpful non-invasive diagnostic technique [26], particularly in the case of deep infiltrates that are not accessible to corneal scrapings, during ongoing anti-parasitic treatment (when trophozoites and cysts may reside in the deeper posterior stroma), and after procedures such as laser-assisted in situ keratomileusis (LASIK), intracorneal ring segments, or radial keratotomy [24,27]. In IVCM, *Acanthamoeba* spp. cysts appear as round or oval (with or without a double-walled aspect) highly refractile structures with a polygonal inner wall and have diameters ranging from 12 to 25 microns [28]. They are sometimes described as existing as part of a typical “starry sky” appearance (Figure 2) [3,12,24,27]. In contrast to the cyst form, the trophozoite form is more difficult to detect using IVCM. They appear as ovoid, S-shaped, or pear-shaped structures within the corneal stroma, but can have a similar appearance to normal corneal keratocytes [8,27,28]. Another possible presentation of trophozoites is the so-called bright “signet ring” [17]. Another typical IVCM finding in AK is a stromal honeycomb pattern of highly reflective activated keratocytes, although this can be a non-specific finding [29]. Serial confocal microscopy can also be useful for monitoring the response to treatment in vivo [26,30,31].

Further diagnostic techniques include PCR, culturing corneal scrapings, and a corneal biopsy. Using PCR for AK diagnosis is becoming more commonly available and has a sensitivity of 84% and a specificity of 100% [32]. Culturing corneal scrapings (positive in 50–74% of patients) [5] and histologic evaluation of stromal biopsy samples (positive in 65% of patients) can also play critical roles in establishing a definitive diagnosis of AK (Table 1) [11,25,32]. However, a corneal culture and a corneal biopsy are limited by a relatively lower sensitivity of the “first attempt” culture or biopsy and their utility can be diminished if there is coinfection with another pathogen [11]. In about 23% of AK cases, a viral, fungal, or bacterial coinfection is present [15], although this number was reported to be as high as 55% in a study by Raghavan et al. [33]. The most common pathogens responsible for coinfection with AK include alpha-hemolytic *Streptococcus* spp., coagulase-negative *Staphylococcus* spp., *Bacillus* spp., *Corynebacterium* spp., *Staphylococcus aureus* spp., and *Streptococcus viridans* spp. [34].

Another emerging tool for early AK diagnosis is anterior segment optical coherence tomography (AS-OCT) [29]. AS-OCT is capable of confirming clinical radial keratoneuritis as highly reflective bands between 20 and 200 µm in length that run obliquely in the corneal stroma, which may then be used to support a diagnosis of AK [29]. However, AS-OCT is not able to identify *Acanthamoeba* spp. cysts or trophozoites [27,29].

Of note, a significant delay in the diagnosis of AK is not uncommon, particularly in several situations: when patients are older than 40 years of age, in non-contact-lens wearers, when IVCM is not performed, and when previous inappropriate medical therapy has been initiated (steroids, antibiotics, antiviral medications) [5,13]. In addition, there is a higher chance of needing a penetrating keratoplasty (PK) in cases where topical steroids have been administered before the AK diagnosis or antimicrobial therapy and when a ring infiltrate is found upon examination [5,36].

### Treatment

The treatment course for AK is often long and challenging, and while the trophozoite form of *Acanthamoeba* spp. is susceptible to multiple therapies, the cystic form is highly drug resistant and may persist for months [8]. The principal initial treatment is the administration of a topical biguanide, such as polyhexamethylene biguanide (PHMB) 0.02–0.08% [37] or chlorhexidine 0.02–0.06% [38], along with or without a topical diamidine, such as propamidine isethionate 0.1% [2,3,4,6,7,8,11,12,20,21,27]. Initially, these medications should be administered every hour around the clock for the first 48–72 h and then tapered gradually. The maintenance therapy of PHMB and propamidine, each 3–4 times per day, is then continued for 4–6 weeks. Both biguanides and diamidines can be toxic to the cornea, often causing corneal epitheliopathy. In cases of toxicity, a decrease in the dosage or allowing for a medication holiday may be required. 

The fact that *Acanthamoeba* spp. has two forms, namely, trophozoites and cysts, has implications for the management strategy in AK. An overarching theme to AK management is that the initial empiric treatment must be aggressive, as trophozoites and immature cysts are significantly more responsive to treatment than mature cysts [11,27]. Topical PHMB and chlorhexidine are effective medications against the cyst form of *Acanthamoeba* spp. [3,11], while propamidine has cystostatic but not cystocidal activity, and thus cannot be used as monotherapy [11,39]. Chlorhexidine 0.02% used as both monotherapy and combination therapy also demonstrated therapeutic efficacy against *Acanthamoeba* spp. without adverse effects [38]. Moreover, low concentrations of benzalkonium chloride (BAK) and povidone iodine seem to exhibit significant antiacanthamoebal activity in vitro [40,41]. In some studies, monotherapy with 0.02% PHMB for the initial AK treatment has been shown to be as effective as combination therapies, including a biguanide plus a diamidine. This therapeutic approach has shown promising cure rates and is an attractive option, as the use of a single medication can improve patient compliance and lower costs as compared to combined therapy [42].

Several other classes of medications have shown promise in the treatment of AK. Systemic antifungal drugs, such as voriconazole and posaconazole, may be useful against the cyst form of *Acanthamoeba* spp., as they inhibit the synthesis of ergosterol, one component of the *Acanthamoeba* spp. cell membrane, making it a potential cystostatic treatment option [18,27]. Miltefosine is an oral medication that is used to treat leishmaniasis and amoebas and has been used in the management of AK. Although at this time it can be expensive and/or difficult to obtain, it has demonstrated efficacy in the treatment of AK [3,12,43]. Other medications, such as topical tea tree oil [1] and neomycin, may also hold promise as accompanying therapies, although prolonged treatment with the latter is not recommended because of its adverse effects on the corneal epithelium, including toxicity and hypersensitivity reactions [2,20]. One important adjunct to topical medications is epithelial debridement, which in addition to providing a tissue sample for culture, can also physically remove trophozoites and cysts limited to the corneal epithelium and enhance topical drug penetration. In cases of intraepithelial infection, epithelium debridement combined with three to four months of antiamoebic treatment may be enough for the successful resolution of disease [2,11,12].

**Figure 3:**
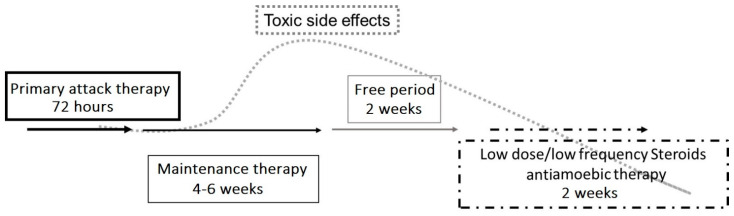
Proposed therapeutic strategy for Acanthamoeba keratitis. An initial aggressive approach to treatment involves hourly topical eye drops (polyhexamethylene biguanide (PHMB) 0.02% and propamidine isethionate 0.1%), followed by tapering to maintenance therapy using PHMB and propamidine 3–4 times per day for 6 weeks. A stable clinical exam after a 2-week antiamoebic free period reduces the risk of medication toxicity and can also unmask the continued presence of trophozoites or cysts. If the infection is still present, the treatment protocol must be repeated. Topical low-dose and low-frequency steroid eye drops (such as loteprednol etabonate and fluorometholone acetate) have been suggested in cases of severe ocular pain, limbitis, or scleritis, and must be used with extreme caution. Topical steroids should only be used with concomitant antiamoebic therapy.

The use of steroids in the treatment of AK remains controversial and should, in general, be used with extreme caution. Steroids can increase the total length of treatment and are known to increase *Acanthamoeba* spp. pathogenicity by promoting the transition of cysts to trophozoites and by increasing trophozoite proliferation [7,8,27]. Nonetheless, steroids are sometimes used in cases of AK when there are intense inflammation and pain out of proportion to what is found in the exam, such as when there is concomitant scleritis and in the presence of deep corneal neovascularization [2,3,4,6,7,8,11,12,20,21,27]. In cases of limbitis and scleritis, steroids have been used to reduce persistent inflammation of the anterior segment and sclera, along with systemic non-steroidal anti-inflammatory drugs (NSAIDs), in order to transition to more long-term immunomodulatory therapy [2,8,11,44]. Early in the course of AK, there is generally no role for steroids, but in cases of prolonged treatment courses with severe corneal inflammation and an inadequate response to topical antiamoebic therapy, topical corticosteroids have been reported to help with the resolution of the disease [3,7,11,27]. AK infection is considered to be eradicated when there is a demonstration of clinical stability after a 2-week suspension of antiamoebic therapy (free period, Figure 3) [11]. Interestingly, anecdotal reports have described the use of topical low-frequency steroids following the suspension of antiamoebic therapy to manage corneal sequelae and also unmask residual amoebic cysts. When used, steroids should always be used with simultaneous antiamoebic coverage.

In cases of AK that are poorly responsive to medical treatment, surgical interventions, including deep anterior lamellar keratoplasty (DALK) or penetrating keratoplasty (PK), may be required. DALK performed within 30–60 days of the onset of symptoms [35] has been shown to be beneficial in eradicating infection in conjunction with antiamoebic treatment before, during, and after surgery, and can yield a statistically significant improvement in final postoperative best-corrected visual acuity (BCVA) compared to preoperative BCVA (average postoperative Snellen visual acuity of approximately 20/25) [45]. While DALK presents less risk of rejection and graft failure when compared with PK, it is relatively technically more difficult and it can be less effective than PK in eradicating the infection, particularly when performed in inflamed eyes or late in the disease course [45].

Full-thickness PK is used to prevent scleral extension and is the most useful and definitive surgical treatment in cases of severe and progressive AK that are unresponsive to medical therapy [15,45]. PK is also indicated in cases of corneal perforation and fulminant corneal abscesses [3,11,27]. However, PK must be performed judiciously as transplants in eyes with severe AK tend to have a poorer prognosis [11]. Grafts must be large enough to remove all affected tissue and minimize the chances of recurrence, but because larger grafts have a higher risk of rejection and failure, surgeons must balance these two factors to appropriately size the corneal transplant. Recurrence occurs most commonly in the first two weeks after surgery, but late recurrences, taking place several months after surgery, also occur [11]. In order to minimize disease recurrence, a good goal to aim for is a 1 mm margin of healthy tissue [39]. In addition, it is recommended to continue antiamoebic treatment for 2–4 weeks following surgery [3,11]. Of note, optical keratoplasties (PK or DALK) that are performed for corneal scarring and irregular astigmatism have better outcomes than therapeutic keratoplasties [42]; therefore, a goal of AK management, whenever possible, should be to utilize medical therapies and delay surgical interventions until *Acanthamoeba* spp. eradication can be achieved. Then, following the resolution of inflammation, perform an optical DALK or PK.

In addition to DALK and PK, amniotic membrane transplantation is an additional procedure that can be used to facilitate complete corneal recovery. In cases of progressive stromal lesions and persistent epithelial defects, amniotic membrane transplantation may be effective in controlling inflammation and promoting stromal and epithelial healing. However, complete stromal and epithelial recovery may require multiple amniotic membrane transplantations and a PK may still be needed [3,4,8,15].

A recent meta-analysis studied the role of photoactivated chromophores for keratitis-corneal cross-linking (PACK-CXL), in addition to standard antimicrobial treatment (SAT), as a therapy for infectious keratitis versus SAT alone [46]. The results showed that in bacterial or fungal keratitis, PACK-CXL may be a useful adjunct therapy for reducing the time to complete corneal healing, but PACK-CXL was not useful compared to SAT alone in reducing the infiltrate size, improving visual acuity, or reducing the risk of adverse effects, such as the worsening of infectious keratitis, corneal melt, or perforations [46]. The microbicidal effect of PACK-CXL likely arises from ultraviolet A (UVA) induced DNA damage and reactive oxygen species release [15,47,48], while pain reduction may be secondary to the suppression of inflammation and nociception by subepithelial nerves [47]. PACK-CXL has been used along with standard antimicrobial therapy to treat persistent cases of *Acanthamoeba* spp. infection [47]; however, there is currently insufficient evidence to support its use in the setting of AK [46,49,50].

## 4. Discussion

### Prognosis and Challenges

Patient quality of life is highly compromised by AK due to severe and persistent ocular pain, long-lasting treatment and healing periods, frequent follow-up visits, and the possible need for complex surgeries that further detract from patients’ personal and professional lives.

Early diagnosis is essential for starting appropriate therapy and, ultimately, to optimize visual outcomes [2,4,27,38]. A late diagnosis of AK correlates with more extensive disease and increases the likelihood of a poorer final visual outcome and the need for a therapeutic PK [5,10,13,45]. Therefore, an early diagnosis is essential for patients, particularly as it pertains to the extent of *Acanthamoeba* spp. involvement, as the earlier the diagnosis, the lower the chance of stromal involvement. In the absence of stromal involvement, the average duration of antiamoebic therapy is around 3–4 months, while in cases of stromal involvement, the treatment period is prolonged and can last more than 8 months with poorer outcomes.

In general, a late diagnosis of *Acanthamoeba* spp. infection is more frequent in non-contact-lens wearers due to a lower index of suspicion [8,11,27]. As many as 90–93% of such cases are initially misdiagnosed as viral, fungal, or bacterial keratitis [8,15,20,22,27]. As an example, in Germany, it has been reported that AK is initially diagnosed as herpetic keratitis in 47.6% of cases, as mycotic keratitis in 25.2% of cases, and as bacterial keratitis in 3.9% of cases [51]. In the same study, a correct diagnosis of AK was found to occur 2.8 ± 4 months (range 0–23 months) after the onset of symptoms [51]. Diagnosing AK is difficult because patients’ presenting symptoms are often nonspecific and classic signs of infection are not always present, particularly in non-contact-lens wearers [4,6,15,27,45]. The interval between the onset of symptoms and the start of effective therapy thus becomes an important predictor of disease outcome [4,5,14,36]. In particular, if the symptom to therapy interval is more than 3 weeks, visual outcomes tend to be poor. However, if the interval is less than 3 weeks, an excellent final visual acuity can be maintained [11,13,27,36].

Another important factor in AK prognosis that is closely related to the delay in diagnosis is disease severity at the time of presentation [4,27,34,36]. Patients with deep stromal involvement or a ring infiltrate (late signs of disease) at the time of presentation have a final visual outcome poorer than 20/25 in 61% of cases, while approximately 87% of patients with early AK at the time of presentation are able to obtain a final visual acuity better 20/25 [36]. When present at the time of diagnosis, indicators of more advanced disease, such as a BCVA worse than 20/50, deep stromal involvement, and a confirmed tissue diagnosis are all associated with a higher chance of eventual PK and a final BCVA < 20/100 [34]. Other prognostic factors that are associated with poor outcomes (corneal perforation, need for PK, duration of antiamoebic treatment >10.5 months, and final BCVA ≤ 20/80) are the presence of severe inflammation and symptom duration >37 days before starting anti-amoebic therapy [3,10,52]. Corticosteroid use and HSV keratitis treatment before anti-amoebic treatment were also found to lead to poorer outcomes [10].

Finally, another prognostic factor is the *Acanthamoeba* spp. genotype. PCR analysis allows for the classification of *Acanthamoeba* spp. into 15 genotype groups defined as T1–T15 [4,53]. Recent evidence suggests that the T4 genotype may be especially virulent [52,53], and this genotype appears to have recently become more widespread in the environment, with increasing drug resistance [3,7,52,53,54].

In summary, AK is a vision-threatening condition that can be challenging to both diagnose and treat. Importantly, a prompt diagnosis reduces the risk of prolonged medical treatment and the need for surgical interventions. Disease severity and the time from symptom onset to diagnosis can predict the duration of treatment, final visual outcomes, and the eventual need for surgery [35]. This information can then be used to counsel patients with respect to their expected treatment timeline and possible sequelae, and patients can then make informed personal and work-related life choices; the hope would be for this information to help improve patient acceptance of their condition, improve compliance, and ultimately, improve outcomes [35]. A standardized and common protocol is still missing in AK management. Up to now, most of our knowledge comes from retrospective case series since there are no randomized, controlled clinical trials to date. The diagnosis of AK can be difficult to make and is generally delayed because of the low specificity of clinical signs and symptoms. Unfortunately, a delayed diagnosis delays appropriate treatment and permits the disease to increase in severity. Accordingly, because of its importance to patient outcomes and quality of life, the early diagnosis of AK represents a critical challenge in the clinical management of this condition.

## Figures and Tables

**Table 1:** Sensitivity and specificity of diagnostic modalities in the diagnosis of Acanthamoeba keratitis.

Diagnostic Test	Sensitivity	Specificity
In vivo confocal microscopy	>90% [7,9,11,33,34]	>90% [9,11,33,34]
Culture of corneal scraping	50–74% [7,13]	100% [35]
Polymerase chain reaction	84% [7,11,35]	100% [11,35]
Histology of stromal biopsy	65% [7,11,34,35]	-
